# TORC1 Regulates Pah1 Phosphatidate Phosphatase Activity via the Nem1/Spo7 Protein Phosphatase Complex

**DOI:** 10.1371/journal.pone.0104194

**Published:** 2014-08-12

**Authors:** Emmanuelle Dubots, Stéphanie Cottier, Marie-Pierre Péli-Gulli, Malika Jaquenoud, Séverine Bontron, Roger Schneiter, Claudio De Virgilio

**Affiliations:** Department of Biology, University of Fribourg, CH-1700 Fribourg, Switzerland; Institute of Biology Valrose, France

## Abstract

The evolutionarily conserved target of rapamycin complex 1 (TORC1) controls growth-related processes such as protein, nucleotide, and lipid metabolism in response to growth hormones, energy/ATP levels, and amino acids. Its deregulation is associated with cancer, type 2 diabetes, and obesity. Among other substrates, mammalian TORC1 directly phosphorylates and inhibits the phosphatidate phosphatase lipin-1, a central enzyme in lipid metabolism that provides diacylglycerol for the synthesis of membrane phospholipids and/or triacylglycerol as neutral lipid reserve. Here, we show that yeast TORC1 inhibits the function of the respective lipin, Pah1, to prevent the accumulation of triacylglycerol. Surprisingly, TORC1 regulates Pah1 in part indirectly by controlling the phosphorylation status of Nem1 within the Pah1-activating, heterodimeric Nem1-Spo7 protein phosphatase module. Our results delineate a hitherto unknown TORC1 effector branch that controls lipin function in yeast, which, given the recent discovery of Nem1-Spo7 orthologous proteins in humans, may be conserved.

## Introduction

Phosphatidate (PA) is a central precursor for membrane phospholipid biosynthesis that also plays regulatory roles in overall lipid metabolism in eukaryotic cells [1], [2]. Evolutionarily conserved PA phosphatases (PAPs) coined lipins dephosphorylate PA to produce diacylglycerol (DAG), which then can be channeled into the synthesis of phospholipids, or be acylated to triacylglycerol (TAG), a major form of fat that is deposited in specialized endoplasmatic reticulum (ER) -derived subcellular structures termed lipid droplets [1], [2], [3], [4], [5]. Underscoring the importance of lipins in this latter process, mutation of mouse lipin-1 causes a near complete absence of TAGs in white adipose tissue and defects in adipocyte differentiation, both common signs of lipodystrophy, while overexpression of mouse lipin-1 promotes obesity [6], [7], [8]. Reminiscent of this phenotype in higher eukaryotes, loss of the single yeast lipin ortholog Pah1 causes, in addition to an overall deregulation of lipid metabolism, a dramatic defect in TAG accumulation when cells are grown to stationary phase [9], [10], [11], [12]. Of note, mammalian and yeast lipins play additional roles in transcriptional modulation of phospholipid biosynthesis genes and may therefore also indirectly contribute to the observed defects in TAG accumulation [13], [14].

Control of lipin function is a tightly regulated process, which depends on the phosphorylation/dephosphorylation of specific residues within lipins that dictate their subcellular localization and/or activity [14]. Pah1, for instance, is phosphorylated at 5 serine residues by the glucose-responsive protein kinase A (PKA), as well as by the cyclin-dependent protein kinase (CDK) Cdc28 and the phosphate-responsive Pho80-Pho85 cyclin-CDK, which collectively (with overlapping specificities) target an additional set of 7 serine/threonine residues [15], [16], [17], [18], [19], [20]. Combined, these phosphorylation events serve to inhibit membrane association and activation of Pah1 and consequently prevent TAG synthesis in cells that proliferate on nutrient rich media [21]. Downregulation of these protein kinases following nutrient starvation, *e.g.* in cells entering stationary phase [9], [11], [22], contributes to the activation of Pah1-driven TAG synthesis. In parallel, Pah1 activation requires the nuclear/ER membrane-associated protein phosphatase Nem1 and its regulatory subunit Spo7, which bind to the acidic carboxy-terminal tail in Pah1 and appear to target most, if not all, of its phosphorylated residues [13], [15], [20], [23], [24]. Nem1-Spo7-mediated dephosphorylation of Pah1, in addition to favoring its membrane association via a N-terminal amphipathic helix, activates the catalytic efficiency of Pah1 and simultaneously primes it for proteasome-dependent degradation [13], [20], [24], [25], [26]. Dephosphorylated Pah1 is therefore both active and particularly unstable, which likely reflects a physiological constraint that requires cells to prevent excess drainage of PA into the synthesis of TAG [21]. Whether the function of the Nem1-Spo7 module is regulated by posttranslational mechanisms is currently not known.

The evolutionarily conserved target of rapamycin complex 1 (TORC1) is at the core of a signaling pathway that controls growth related processes such as protein, lipid, and nucleotide metabolism in response to diverse signals including growth hormones (insulin/IGF), energy/ATP levels, and amino acids [27], [28]. Deregulation of TORC1 is associated with various pathological conditions in humans including cancer, obesity, type 2 diabetes and neurodegeneration [29]. Among other substrates (*e.g.*, S6 kinase, 4E-binding protein, TFEB, and ULK1), the essential serine/threonine protein kinase within mammalian TORC1 also directly phosphorylates, and thereby inhibits the function of lipin-1 [30], [31], [32], [33], [34]. Whether TORC1-mediated control of lipin function represents an ancestral mechanism that regulates TAG accumulation remains to be determined. Here, we show that TORC1 inhibition in yeast induces dephosphorylation and activation of Pah1, which is accompanied by Pah1-dependent accumulation of TAG. Moreover, we demonstrate that all of these processes depend on the presence of the Nem1-Spo7 module and that TORC1 specifically regulates the phosphorylation status of Ser^195^ within Nem1 to properly regulate Pah1.

## Materials and Methods

### Strains, Plasmids, and Growth Conditions

Yeast cells were pre-grown overnight at 30°C in standard rich medium with 2% glucose (YPD) or synthetic defined (SD) medium with 2% glucose and supplemented with the appropriate amino acids for maintenance of plasmids. Prior to the experiments, cells were diluted to an OD_600_ of 0.1 in YPD and grown until they reached an OD_600_ of 0.6–0.8. Rapamycin was dissolved in 10% Tween-20/90% ethanol and used at a final concentration of 200 ng ml^−1^. Strains and plasmids used in this study are listed in Tables 1 and 2, respectively. All tagged proteins studied were functional and expressed from their own promoter except in the case of Nem1-PtA and Spo7-myc_13_ (used for phosphopeptide analyses by mass spectrometry [MS]), which were expressed under the control of the galactose-inducible *GAL1* promoter. For galactose induction of the expression of the respective genes, cells were pre-grown on SD medium with 2% raffinose and 0.1% sucrose, diluted to an OD_600_ of 0.1 with the same medium, and grown for 3 h in the presence of 2% galactose.

**Table 1:** Strains Used in This Study.

Strain	Genotype	Source	Figure
BY4742	*MAT*α; *his3*Δ*1, leu2*Δ*0, ura3*Δ*0, lys2*Δ*0*	Euroscarf	1A–D, 4A
ED19-4B	*MAT*α; *his3*Δ*1, leu2*Δ*0, ura3*Δ*0, pah1*Δ*::kanMX4*	This study	1A/B/D
ED28-9A	[BY4742] *nem1*Δ*::kanMX4*	This study	1A/B, 3A–C, 4B
ED29-1A	[BY4742] *spo7*Δ*::kanMX4*	This study	1A
YOR245C	[BY4742] *dga1*Δ*::kanMX4*	Euroscarf	1A
YNR008W	[BY4742] *lro1*Δ*::kanMX4*	Euroscarf	1A
RSY3290	[BY4742] *dga1*Δ*, lro1*Δ*::kanMX4*	This study	1A
ED65-1C	*MAT*α; *his3*Δ*1, leu2*Δ*0, ura3*Δ*0, app1*Δ*::HIS3MX6, lpp1*Δ*:: kanMX4, dpp1*Δ*::kanMX4*	This study	1C/D
ED67-5C	[ED65-1C] *nem1*Δ*::kanMX4*	This study	1C/D
ED2545	[BY4742] *PAH1-HA_3_::kanMX4*	This study	1E, 2A/C
ED41-8B	*MAT*α; *his3*Δ*1, leu2*Δ*0, ura3*Δ*0, nem1*Δ*::kanMX4, PAH1-HA_3_::kanMX4*	This study	1E, 2A/C/D, 4C/D
ED36-11C	[BY4742] *nem1*Δ*::kanMX4, spo7*Δ*::kanMX4, PAH1-HA_3_::kanMX4*	This study	2B–D
ED36-5D	[BY4742] *nem1*Δ*::kanMX4, spo7*Δ*::kanMX4*	This study	MS analyses
NMY51	*MAT* a; *his3*Δ*200, trp1-901, leu2-3,112, ade2, LYS::(lexAop)_4_-HIS3, ura3::(lexAop)_8_-lacZ, ade2::(lexAop)_8_-ADE2, GAL4*	Dual-systems	2E

**Table 2:** Plasmids Used in This Study.

Plasmid	Genotype	Source	Figure
YCplac111	*CEN/ARS*, *LEU2*	[54]	2C/D
YCplac33	*CEN/ARS, URA3*	[54]	
pRS416	*CEN/ARS, URA3*	[55]	
pRS415	*CEN/ARS, LEU2*	[55]	4C/D
pSB2235	[YCplac111] *NEM1-HA_3_*	This study	2B/D, 3A/B, 4A/B
pED2321	[YCplac111] *DGA1-PtA*	This study	2A
p2202	[YCplac111] *NEM1-PtA*	[13]	2A/C, 3C, 4C/D
pED2378	[YCplac111] *NEM1^S195A^-PtA*	This study	4C/D
pSB2411	[YCplac111] *GAL1p-SPO7-myc_13_*	This study	MS analyses
pSB2413	[pRS416] *GAL1p-NEM1-PtA*	This study	MS analyses
pED2342	[YCplac33] *SPO7-PtA*	This study	2B/D
pED2520	[YCplac33] *DGA1-PtA*	This study	2D
pPR3-N	2*μ*, *NUBG-HA, TRP1*	Dualsystems	
pCab	*CEN*, *CUB-LEXA, LEU2*	Dualsystems	
pMJA2383	[pPR3-N] *NUBG-HA-SPO7*	This study	2E
pMJA2381	[pPR3-N] *NUBG-HA-NEM1*	This study	2E
pMJA1854	[pCab] *MON1-CUB-LEXA*	This study	2E
pMJA2379	[pCab] *NEM1-CUB-LEXA*	This study	2E
pMJA2387	[pCab] *SPO7-CUB-LEXA*	This study	2E
pMJA2389	[pCab] *PAH1-CUB-LEXA*	This study	2E
pSB2364	[YCplac111] *NEM1^S195A^-HA_3_*	This study	4B
pSB2353	[YCplac111] *NEM1_1-249_-HA_3_*	This study	4A
pSB2586	[YCplac111] *NEM1_88-446_-HA_3_*	This study	4A
pSB2587	[YCplac111] *NEM1_147-446_-HA_3_*	This study	4A
pSB2588	[YCplac111] *NEM1_199-446_-HA_3_*	This study	4A
pSB2585	[YCplac111] *NEM1_250-446_-HA_3_*	This study	4A

### Protein Analyses

Total protein extracts were prepared as previously described [35]. SDS-PAGE and immunoblot analyses were performed according to standard protocols. For the analysis of protein phosphorylation states, we used the previously described method for Phos-tag acrylamide gel electrophoresis [36]. Mouse anti-HA (12CA5) antibodies and purified IgG from rabbit serum (Sigma) were used at concentrations of 1 µg ml^−1^. Mouse anti-phosphoglycerate kinase 1 antibodies (Invitrogen) and rabbit anti-Adh1 antibodies were used at dilutions of 1∶5000 and 1∶200000, respectively. Horseradish peroxidase-conjugated goat anti-mouse/anti-rabbit antibodies (Biorad) or goat anti-mouse/mouse anti-rabbit IgG light chain specific antibodies (Jackson Immunoresearch) were used at a 1∶5000 dilution.

### Protein A Pulldown and Two-Hybrid Experiments

Exponentially growing cells treated, or not, with rapamycin and expressing the indicated Protein A (PtA) fusion proteins were harvested at 4°C by centrifugation, washed once with H_2_O and resuspended in lysis buffer (150 mM KCl, 20 mM Tris HCl pH 8.0, 5 mM MgCl_2_, 1% Triton X-100, 1 mM PMSF, 1x EDTA free protease inhibitor cocktail [Roche], and 1x PhosSTOP phosphatase inhibitor cocktail [Roche]) and frozen in liquid nitrogen. Lysates were prepared by disruption of frozen cells with glass beads (0.5 mm diameter) using a Precellys cell disruptor and subsequent clarification by centrifugation (5 min at 14000 rpm; 4°C). Protein concentrations in lysates were determined by the method of Bradford [37]. Cleared lysates were incubated with prewashed IgG sepharose beads (GE Healthcare) for 2 h at 4°C and washed 3 times with 5 volumes of lysis buffer. Immunoprecipitates were resuspended in 2× Laemmli buffer, denatured for 10 min at 65°C, and used for SDS-PAGE and immunoblot analyses. The split-ubiquitin membrane based two-hybrid system was used essentially as described [38].

### *In vitro* Dephosphorylation of Nem1

*In vitro* dephosphorylation of Nem1 was performed after immunoprecipitation of Nem1-PtA from yeast cell extracts. Cells (50 OD_600_) were treated with 6% TCA and disrupted with glass beads in 500 µl urea Buffer (50 mM Tris HCl pH 7.5, 5 mM EDTA, 6 M urea, and 1% SDS, 1x PhosSTOP phosphatase inhibitor cocktail and 1x EDTA free protease inhibitor cocktail. Cell extracts were then diluted in 4 ml lysis buffer and immunoprecipitation was performed as described above. Samples were then washed 3 times with lysis buffer and resupended in 100 µl of FastAP buffer (10 mM Tris HCl pH 8.0, 0.1 M KCl, 0.02% Triton X-100, 0.1 mg/ml BSA, 5 mM MgCl_2_, and 1x protease inhibitor cocktail) containing, or not, 5 units of FastAP phosphatase (Fermentas) with or without phosphatase inhibitor cocktail. Reactions were incubated for 30 min at 37°C and terminated by addition of 2x SDS-PAGE loading buffer. After 10 min at 65°C, the samples were resolved by phosphate affinity SDS-PAGE on Phos-tag gels and immunoblotted with anti-IgG antibodies.

### Identification of Nem1 Phosphopeptides by Mass Spectrometry

To identify phosphorylation sites in Nem1, overexpressed Nem1-PtA was purified from *nem1*Δ *spo7*Δ double mutants overexpressing Spo7-myc_13_. Cells were grown exponentially on galactose-containing medium and then subjected, or not, to a 60-min rapamycin treatment. Following affinity purification, Nem1-PtA was separated by SDS-PAGE and the band corresponding to Nem1-PtA was excised and digested with trypsin and chymotrypsin. Phosphopeptides were enriched via an affinity step on TiO_2_ micro-columns. To identify phosphorylated peptides, the eluates from the TiO_2_ columns were analyzed by nano-LC-MS/MS.

### Lipid Labeling

*In vivo* TAG synthesis was monitored by labeling the neutral lipid pool with radioactively labeled palmitic acid (10 µCi ml^−1^ [9,10-^3^H]palmitic acid; American Radiolabeled Chemicals) that was added to exponentially growing cells just prior to their treatment with rapamycin or vehicle for 90 min. Cells (15 OD_600_) were collected and lipids were extracted with chloroform/methanol (1∶1), separated by thin-layer chromatography (TLC) on silica gel 60 plates (Merck, Darmstadt, Germany), developed in petroleum-ether/diethyl-ether/acetic acid (70∶30∶2), and quantified using a Berthold Tracemaster 20 Automatic TLC-Linear Analyzer (Berthold Technologies, Bad Wildbach, Germany).

### Enzymatic measurement of [DAG+TAG] levels

Cells (30–50 OD_600_) growing exponentially on YPD medium were treated with rapamycin or vehicle, harvested by centrifugation at 4°C, washed once with H_2_O, resupended in 300 µl of extraction buffer (50 mM Tris HCl pH 7.5, 0.3% Triton X-100), and lysed with glass beads using a Precellys cell disruptor. The total lysates were clarified by centrifugation (5 min at 5000 rpm) and lipids were extracted from these lysates [39]. The MBL Triglyceride Quantification Kit (JM-K622-100) was used to quantify the [DAG+TAG] levels after resuspension of the dried lipid extracts in the assay buffer provided with the kit.

### Phosphatidate phosphatase activity assay

PAP activity was determined in cell lysates by measuring the formation of fluorescent DAG from NBD-PA (1-acyl-2-{12-[(7-nitro-2-1,3-benzoxadiazol-4-yl)amino]dodecanoyl}-*sn*-glycero-3-phosphate ammonium salt) (Avanti® Polar lipids, Inc). Protein lysates were essentially obtained as described in the Protein A pulldown section, except that the buffer used was 50 mM Tris HCl pH 8.0, 0.5 mM PMSF, 10 mM β-mercaptoethanol, 1x EDTA free protease inhibitor cocktail, and 1x PhosSTOP phosphatase inhibitor cocktail, and lysates were clarified by centrifugation for 5 min at 1600 rpm. Reactions (100 µl; 80 µg of total protein extract) were carried out in buffer containing 50 mM Tris HCl pH 8.0, 1 mM MgCl_2_ or 4 mM EDTA, and 10 mM β-mercaptoethanol, and started by the addition of NBD-PA (2 mM) solubilized in 10 mM Triton X-100. The reactions were incubated for 15 min at 30°C and terminated by the addition of 0.5 ml of 0.1 M HCl (in methanol). Following addition of 1 ml chloroform and 1 ml of 1 M MgCl_2_ and centrifugation (1000 rpm for 10 min at room temperature), the chloroform-soluble lipid fractions were isolated and dried under nitrogen as described [40]. Lipids were resuspended in chloroform/methanol (1∶1), deposited on silica gel 60 plates (Merck) and separated by TLC using chloroform/methanol/H_2_O (62∶25∶4) as a solvent system [41]. NBD-DAG production was determined by recording fluorescence with a Typhoon FLA 9500 device (excitation 473 nm; Filter BPB1, centered at 530 nm; width 20 nm) and quantified with the ImageQuantTLsoftware (GE Healthcare).

## Results and Discussion

### TORC1 inhibition activates Pah1 phosphatidate phosphatase via the Nem1-Spo7 protein phosphatase module

**Figure 1:**
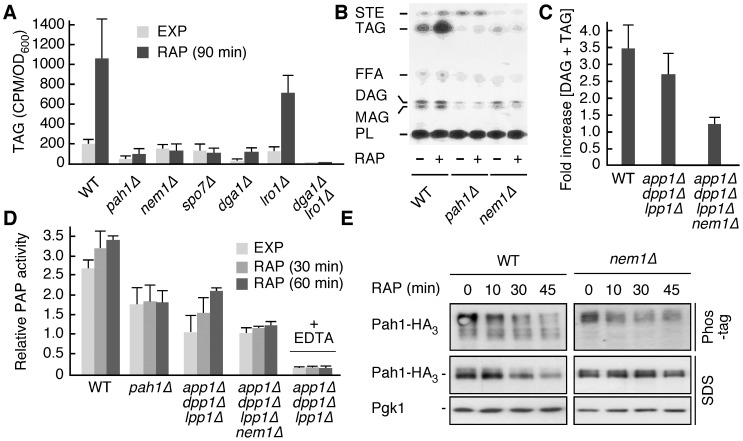
TORC1 inhibition activates Pah1 phosphatidate phosphatase via the Nem1-Spo7 protein phosphatase module. (A) Incorporation of radioactively labeled palmitic acid into triacylglycerol (TAG) was monitored in exponentially growing (EXP) and rapamycin-treated (RAP; 90 min) cells. Relevant genotypes of strains are indicated (WT, wild type). (B) Representative TLC plate showing radioactively-labeled, separated lipid samples from the experiment in (A) that were extracted from exponentially growing (RAP; −) and rapamycin-treated (RAP; +) WT, *pah1*Δ, and *nem1*Δ strains. STE, steryl esters; FFA, free fatty acids; DAG, diacylglycerol; MAG, monoacylglycerol; PL, phospholipids. (C) The combined levels of DAG and TAG were determined in rapamycin-treated (4 h) cells using a commercially available enzymatic kit and expressed in each case relative to the respective levels in exponentially growing cells. (D) Relative PAP activity in exponentially growing (EXP) and rapamycin-treated (RAP; 30 min and 60 min) cells. Results are presented as relative activities compared to the activity in exponentially growing *app1*Δ *dpp1*Δ* lpp1*Δ cells (defined as 1.0), which express Pah1 as only source of PAP activity [44]. Assays carried out in the presence of EDTA are indicated (+ EDTA). (E) Phos-tag phosphate-affinity gel electrophoresis and SDS-PAGE analyses of endogenously tagged Pah1-HA_3_ in exponentially growing WT and *nem1*Δ cells treated with rapamycin (RAP) for the indicated times. The levels of Pgk1 served as loading controls. In Figures 1A, 1C, and 1D, each bar represents the mean ± SD of three experiments.

To address the question whether TORC1 regulates TAG synthesis in yeast, we treated wild-type and various mutant cells with rapamycin and assessed the *in vivo* incorporation of [^3^H]palmitic acid into TAG. TORC1 inhibition resulted in a significant (>5 fold) increase of TAG levels in wild-type, but not in *pah1*Δ cells (Fig. 1A and 1B). Interestingly, rapamycin-induced TAG synthesis further required the acyl-CoA:diacylglycerol acyltransferase Dga1, which forms TAG from acyl-CoA and DAG [42], but not the phospholipid:diacylglycerol acyltransferase Lro1 [43], which forms TAG via transesterification of fatty acids from phospholipids to DAG (Fig. 1A). An independent enzymatic assay confirmed that TORC1 inhibition resulted in roughly a 3.5-fold increase of the cellular levels of DAG and TAG combined, and that this increase depended mainly on Pah1, but not on any of the three other known PAP enzymes in yeast (*i.e.* App1, Dpp1, and Lpp1; Fig. 1C) [9], [44], [45], [46], [47]. In line with these data, the relative PAP activity of Pah1 increased more than 2-fold in *app1*Δ *dpp1*Δ* lpp1*Δ cells after a 1-h rapamycin treatment, while the basal PAP activity in *pah1*Δ cells provided by App1, Dpp1, and Lpp1 combined remained unaffected by the same treatment (Fig. 1D). Importantly, the addition of EDTA, which chelates the Mg^2+^ required for Pah1 activity [9], [12], abolished Pah1 activation in *app1*Δ *dpp1*Δ* lpp1*Δ cells following rapamycin treatment (Fig. 1D). Together, these data show that inactivation of TORC1 results in activation of Pah1 activity and Dga1-dependent channeling of DAG into TAG.

Consistent with a suggested role for the Nem1-Spo7 protein phosphatase in Pah1 activation in cells approaching stationary phase [15], [20], [21], our analyses revealed that Nem1 was required for the activation of Pah1 in rapamycin-treated *app1*Δ *dpp1*Δ* lpp1*Δ cells (Fig. 1D). Consequently, loss of Nem1 (or of Spo7) rendered cells unable to synthesize and accumulate TAGs when treated with rapamycin (Fig. 1A, 1B, and 1C). Notably, as seen in cells approaching stationary phase [15], in rapamycin-treated wild-type cells activation of Pah1 correlated with both its dephosphorylation (as visualized on Phos-tag phosphate affinity gel electrophoresis) and degradation (Fig. 1E). Loss of Nem1, however, prevented the dephosphorylation, activation, and degradation of Pah1 under the same conditions (Fig. 1D and 1E).

### TORC1 moderately impacts on the interaction between Pah1 and the Nem1-Spo7 module

**Figure 2:**
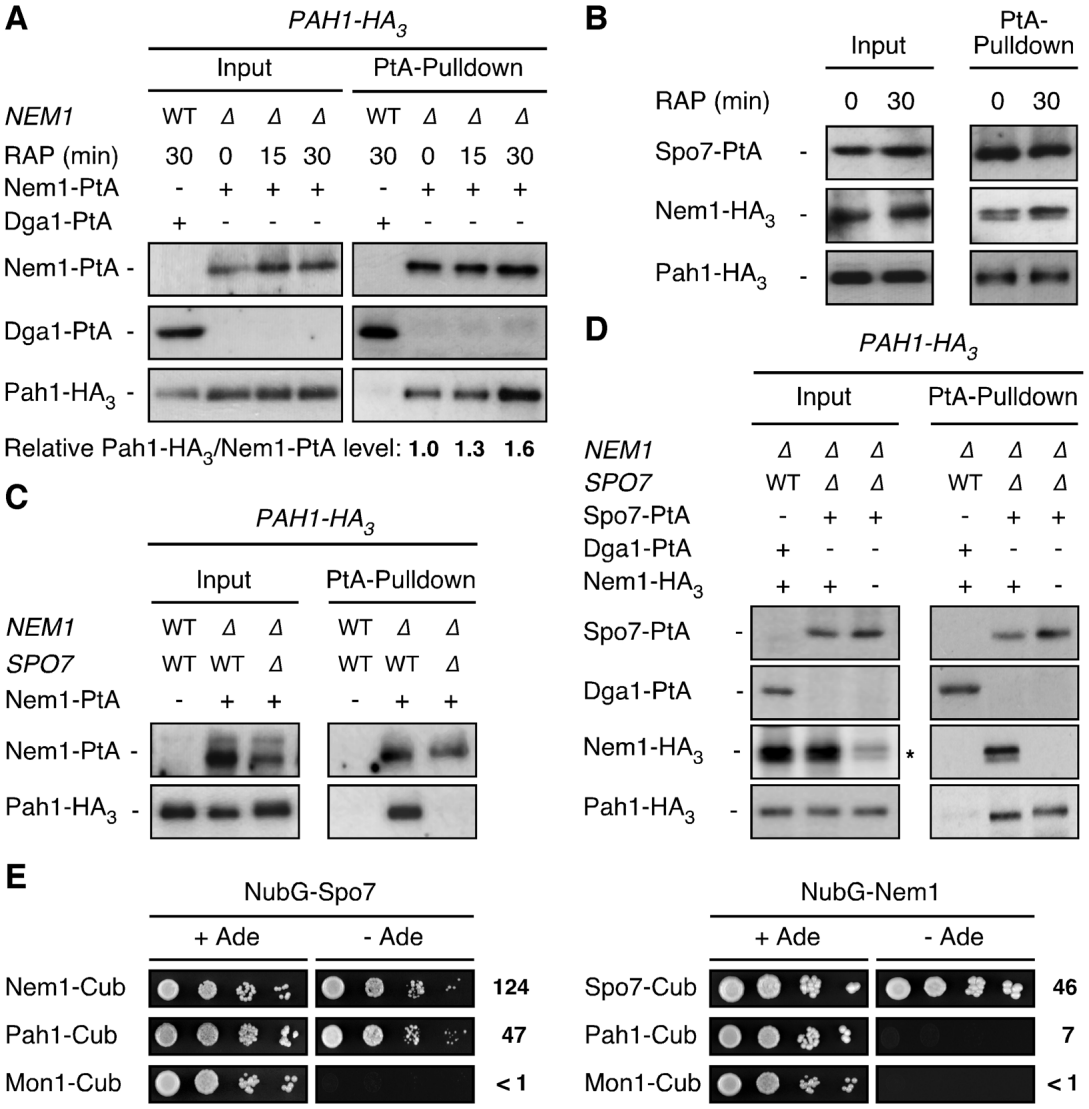
TORC1 has little impact on the interaction between Pah1 and the Nem1-Spo7 module. (A) Biochemical interaction between Nem1 and Pah1. Plasmid-encoded Dga1-PtA or Nem1-PtA was immunoprecipitated from extracts of Pah1-HA_3_-expressing wild-type (lane 1) or *nem1*Δ cells (lanes 2–4), respectively, that were either grown exponentially (0 min) or treated with rapamycin (RAP) for the indicated times. Lysates (Input) and immunoprecipitates (PtA-Pulldown) were subjected to SDS-PAGE and immunoblots were probed with anti-HA or anti-IgG antibodies. WT and Δ denote wild-type and deleted version of *NEM1*, respectively. Numbers below the PtA-Pulldown blots indicate the relative amount of Pah1-HA_3_ that bound to and was pulled down with Nem1-PtA (normalized to the samples of exponentially growing cells). (B) Biochemical interaction between Spo7 and Nem1/Pah1. Plasmid-encoded Spo7-PtA was immunoprecipitated from extracts of untreated (0 min) and rapamycin-treated (RAP; 30 min) *nem1*Δ *spo7*Δ *PAH1-HA_3_* cells that coexpressed plasmid-encoded Nem1-HA_3_. For details see (A). (C) The interaction between Nem1 and Pah1 requires Spo7. Plasmid-encoded Nem1-PtA was immunoprecipitated from extracts of exponentially growing, Pah1-HA_3_-expressing *nem1*Δ (lane 2) or *nem1*Δ *spo7*Δ (lane 3) cells. Pah1-HA_3_-expressing wild-type cells were used as control (lane 1). Please note that loss of Spo7 consistently resulted in decreased levels of Nem1. For details see (A). WT and Δ denote wild-type and deleted version(s), respectively, of the indicated gene(s). (D) The interaction between Spo7 and Pah1 does not require Nem1. Plasmid-encoded Dga1-PtA or Spo7-PtA was immunoprecipitated from extracts of exponentially growing, Pah1-HA_3_-expressing *nem1*Δ (lane 1) or *nem1*Δ* spo7*Δ (lanes 2 and 3) cells, which coexpressed, or not, plasmid-encoded Nem1-HA_3_. Please note that our anti-HA antibodies weakly cross-react with proteins that are present in cell lysates (indicated by the asterisk), but absent in the PtA-pulldown fractions. For details see (A). WT and Δ denote wild-type and deleted version(s), respectively, of the indicated gene(s). (E) Spo7 specifically interacts with both Nem1 and Pah1, while Nem1 only interacts with Spo7, but not with Pah1, when assayed in a split-ubiquitin membrane-based yeast two-hybrid assay. Interactions were tested by monitoring either growth on plates lacking adenine (-Ade), or β-galactosidase activities (in Miller units; numbers on the right of the panels represent the means of three independent experiments performed with exponentially growing cells) of cells expressing the indicated combinations of NubG-Spo7 or NubG-Nem1 and Nem1-Cub, Pah1-Cub, Spo7-Cub, or Mon1-Cub (control).

Based on these results, we considered it possible that Nem1-Spo7, rather than acting as a passive module that counteracts the activities of various protein kinases (*e.g.*, Cdc28, Pho80-Pho85, and PKA), may in fact be part of a specific TORC1-controlled regulatory signaling branch. To address this issue experimentally, we analyzed the predicted *in vivo* interactions between Nem1-Spo7 and Pah1 using classical co-immunoprecipitation (co-IP) assays in exponentially growing and rapamycin-treated cells. Nem1-PtA, but not a control protein (Dga1-PtA), interacted with HA_3_-tagged Pah1 (Fig. 2A; and data not shown). Rapamycin treatment caused a slight increase of the Nem1-PtA protein levels and moderately enhanced the relative amount of Pah1-HA_3_ that was co-IPed with Nem1-PtA (*i.e.* 1.55-fold after a 30-min rapamycin treatment; SD ±0.29; n = 4; Fig. 2A). In parallel experiments, Nem1-HA_3_ and Pah1-HA_3_ also robustly co-IPed with Spo7-PtA in both exponentially growing and rapamycin-treated cells (Fig. 2B). From these results, we infer (i) that the Nem1-Spo7 module constitutively binds a fraction of Pah1, which is also in line with a previous report that implicated the Pah1 carboxy-terminal acidic domain in mediating the interaction with Nem1-Spo7 in exponentially growing cells [48], and (ii) that TORC1 does not play a major role in controlling the protein-protein interactions among Nem1, Spo7, and Pah1. Interestingly, in this context, we further observed that the Nem1-Pah1 interaction (in both exponentially growing and rapamycin-treated cells) was entirely dependent on the presence of Spo7 (Fig. 2C; and data not shown), while the Spo7-Pah1 interaction did not require Nem1 (Fig. 2D; and data not shown). Thus, Nem1 binds and targets Pah1 indirectly via Spo7. In further support of this conclusion, Spo7 interacted with both Nem1 and Pah1, while Nem1 only interacted with Spo7, but not with Pah1, when examined in a split-ubiquitin, membrane-based two hybrid assay (Fig. 2E). These findings therefore also provide a rationale for our observation that loss of Spo7 phenocopies the loss of Nem1 with respect to the defect in rapamycin-induced TAG synthesis (Fig. 1A).

### TORC1 inhibits Pah1 function in part by controlling the phosphorylation status of Ser^195^ in Nem1

**Figure 3:**
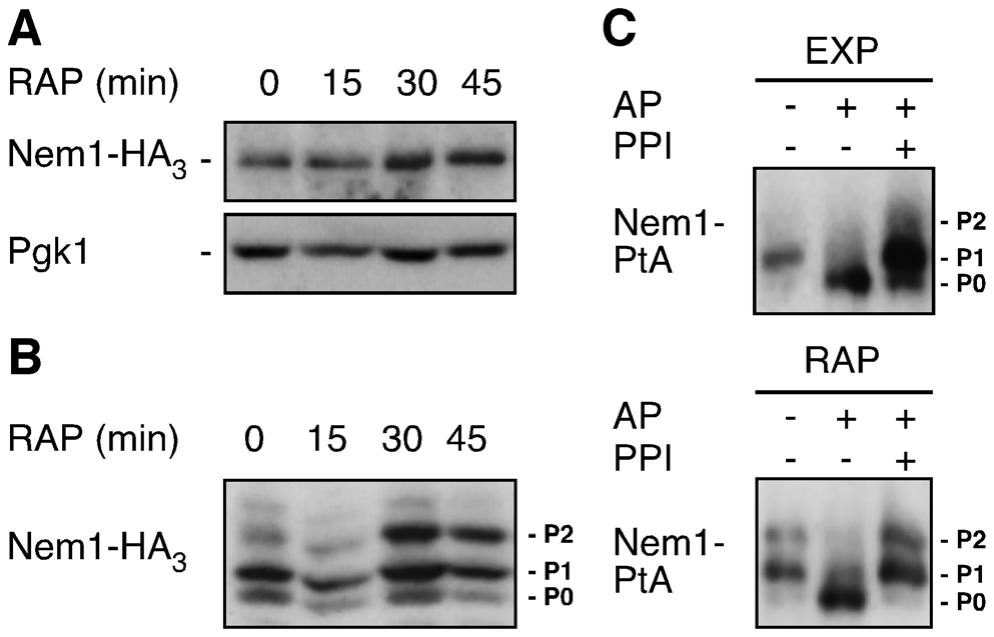
TORC1 antagonizes Nem1 phosphorylation. (A, B) SDS-PAGE (A) and Phos-tag phosphate-affinity gel electrophoresis (B) analyses of plasmid-encoded Nem1-HA_3_ in exponentially growing *nem1*Δ cells treated with rapamycin (RAP) for the indicated times. The levels of Pgk1 served as loading controls in (A). (C) Phosphorylation pattern analysis of Nem1-PtA on Phos-tag gels. Plasmid-encoded Nem1-PtA was purified from exponentially growing (EXP) and rapamycin-treated (RAP; 30 min) *nem1*Δ cells and treated with (+), or without (−), alkaline phosphatase (AP) in the absence (−), or presence (+), of phosphatase inhibitors (PPI). P0, P1, and P2 (in B and C) denote 3 differentially phosphorylated Nem1-HA_3_ or Nem1-PtA isoforms.

To address the possibility that TORC1 may control the function of the Nem1-Spo7 module via posttranslational modification(s), we analyzed the potential phosphorylation patterns of Nem1 and Spo7 in exponentially growing and rapamycin-treated cells using Phos-tag phosphate affinity gel electrophoresis. When extracted from exponentially growing or rapamycin-treated cells, Nem1-HA_3_ migrated as a single band following analysis by standard SDS PAGE (Fig. 3A). Phos-tag phosphate affinity gel electrophoresis, in contrast, revealed two major Nem1-HA_3_ (or Nem1-PtA) isoforms (labeled P0 and P1) in extracts from exponentially growing cells and an additional third major isoform in extracts from rapamycin-treated cells (labeled P2; Fig. 3B and 3C; and data not shown). Alkaline phosphatase (AP) treatment of purified Nem1-PtA from exponentially growing or rapamycin-treated cells converted the Nem1-PtA P1 or P1/P2 isoforms, respectively, to the P0 isoform, unless protein phosphatase inhibitor cocktail (PPI) was added prior to the addition of AP (Fig. 3C). Thus, Nem1 appears to harbor at least one amino acid residue that is constitutively phosphorylated and one residue that is specifically phosphorylated following TORC1 inactivation. Since similar analyses with Spo7 did not readily reveal any potential phosphorylation events (data not shown), we focused our studies on the identification of the two amino acid residues in Nem1 that we assumed are phosphorylated *in vivo*. Using a combination of MS and tandem MS (TMS) analyses on Nem1-PtA samples that had been purified from exponentially growing and rapamycin-treated cells, we identified several serines (at positions 51, 140, 143, 150, 151, 157, 158, 208, 210, and 212) that are potentially phosphorylated. None of these serine residues, however, appeared to be differentially phosphorylated between the samples subjected to MS-TMS analyses and only Ser^210^, which has already previously been identified [49], received a high confidence phosphorylation score.

**Figure 4:**
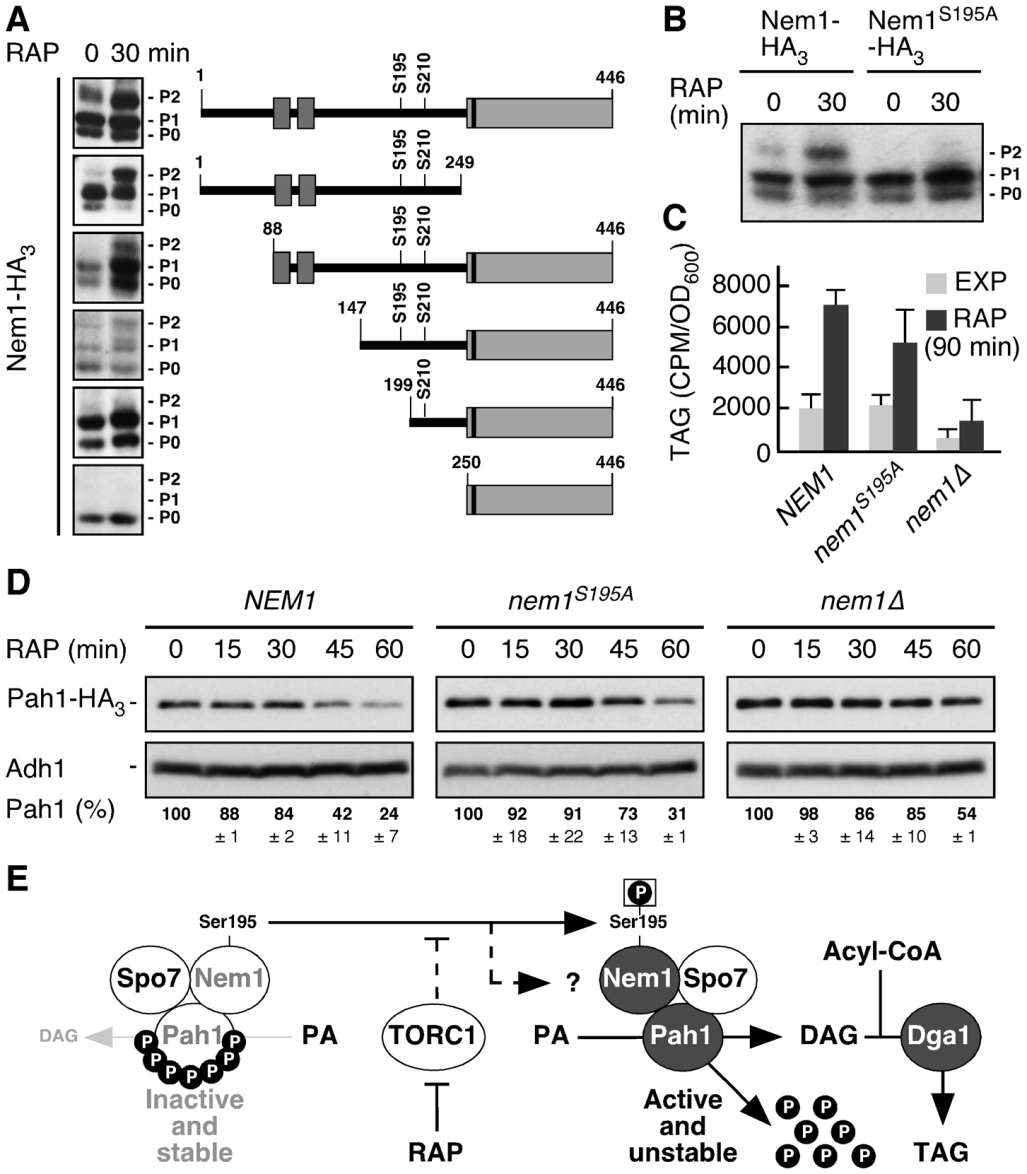
TORC1 inhibits Pah1 function in part by preventing phosphorylation of Ser^195^ in Nem1. (A) Phos-tag phosphate-affinity gel electrophoresis analysis of full length and schematically indicated truncated, plasmid-encoded Nem1-HA_3_ variants in exponentially growing (RAP; 0 min) and rapamycin-treated (RAP; 30 min) wild-type cells. The two dark grey boxes in the N-terminal region denote membrane-spanning regions and the black stripe within the highly conserved C-terminal domain (grey box) indicates the position of the Nem1 catalytic site. (B) Phos-tag phosphate-affinity gel electrophoresis analysis of plasmid-encoded Nem1-HA_3_ and Nem1^S195A^-HA_3_ in exponentially growing (RAP; 0 min) and rapamycin-treated (RAP; 30 min) *nem1*Δ cells. P0, P1, and P2 denote 3 differentially phosphorylated full-length (in [A] and [B]) or truncated (in [A]) Nem1-HA_3_ isoforms. (C) Incorporation of radioactively labeled palmitic acid into triacylglycerol (TAG) was monitored in exponentially growing (EXP) and rapamycin-treated (RAP; 90 min) *nem1*Δ *PAH1-HA_3_* cells that carried either an empty plasmid or a plasmid allowing the expression of PtA-tagged Nem1 or Nem1^S195A^. Relevant genotypes of strains are indicated. (D) SDS-PAGE analysis of endogenously tagged Pah1-HA_3_ from *nem1*Δ cells coexpressing, or not, plasmid-encoded PtA-tagged Nem1 or Nem1^S195A^. Cells were either grown exponentially (RAP; 0 min) or treated with rapamycin (RAP) for the times indicated. Pah1-HA_3_ levels were quantified, normalized with respect to the Adh1 loading control, and expressed in percent relative to the value at time point 0 (see numbers below the panels). Numbers represent means ± SD of three experiments. Relevant genotypes of strains are indicated. (E) Model for the role of TORC1 in controlling TAG synthesis in yeast. TORC1 indirectly regulates (dashed bar) the phosphorylation status of Ser^195^ (and potentially other residues; indicated by the dashed arrow and the question mark) in Nem1 by activating or inhibiting hitherto unknown protein phosphatase(s) or kinase(s), respectively. Arrows and bars denote positive and negative interactions, respectively. For details, see text.

To identify the TORC1-controlled residue in Nem1 that appears to have escaped detection by our MS analyses, we analyzed the phosphorylation pattern of a series of truncated forms of Nem1-HA_3_ in exponentially growing and rapamycin-treated cells. Only Nem1-HA_3_ variants containing amino acids 147 to 199 of Nem1 displayed on Phos-tag gels the additional rapamycin-induced isoform that seemed to correspond to the P2 isoform observed with full-length Nem1-HA_3_ (Fig. 4A). Individual mutations of the Ser/Thr residues to Ala within this domain allowed us to pinpoint Ser^195^, which, when mutated to Ala, specifically prevented the formation of the rapamycin-induced P2 isoform of the respective Nem1^S195A^-HA_3_ variant (Fig. 4B). Interestingly, the remaining P1 isoform displayed by Nem1^Ser195^-HA_3_, whether it was extracted from exponentially growing or from rapamycin-treated cells, further collapsed into one single P0 isoform when also Ser^210^ was mutated to Ala (Fig. 4A; and data not shown). A fraction of Nem1-HA_3_ is therefore constitutively phosphorylated at Ser^210^, while the phosphorylation of Ser^195^ specifically requires downregulation of TORC1.

To assess whether the phosphorylation of Ser^195^ in Nem1 is functionally relevant for Pah1 activation and subsequent TAG accumulation, we first measured TAG accumulation in rapamycin-treated *nem1*Δ cells expressing plasmid-encoded, PtA-tagged Nem1 or Nem1^S195A^. In these experiments, TAG levels were on average slightly reduced in rapamycin-treated Nem1^S195A^-expressing cells when compared to Nem1 expressing cells (Fig. 4C). In these *in vivo* assays, however, it is possible that potentially more significant effects of the *nem1^S195A^* allele on TAG levels were masked by the activities of TAG lipases (*e.g.*, Tgl3, Tgl4, or Tgl5), which may also be implicated in homeostatic control of TAG levels in rapamycin-treated cells. To further assess the functional relevance of Ser^195^ in Nem1, we therefore also measured the kinetics of Pah1 degradation in rapamycin-treated cells, which is a sensitive proxy for the *in vivo* function of Nem1 (Fig. 1E). In this assay, expression of Nem1^S195A^ significantly compromised, although not as strongly as loss of Nem1, the degradation of Pah1 (Fig. 4D). Notably, Nem1^S195A/S210A^-HA_3_ and Nem1^S195A^-HA_3_ expression similarly abrogated Pah1 degradation in rapamycin-treated cells, which indicates that Ser^210^ phosphorylation is not part of a TORC1-controlled mechanism (data not shown). In sum, we infer from our current results that TORC1 restrains Nem1 function in part by favoring the dephosphorylated state of Ser^195^ in Nem1 as well as in part by still elusive mechanisms, which may formally also implicate additional phosphorylation sites within Nem1 (or Spo7) that have escaped our current analyses (Fig. 4E). Our data therefore identify the Nem1-Spo7 module as an element of a hitherto unknown TORC1 effector branch controlling lipin function in yeast and highlight that the function of the Nem1-Spo7 module, like the one of the respective counterbalancing protein kinases, is fine-tuned by regulatory processes.

Like Pah1, lipin-1 is specifically dephosphorylated by the human Nem1 orthologous C-Terminal Domain Nuclear Envelope Phosphatase 1 (CTDNEP1; aka dullard) [50], [51]. Moreover, CTDNEP1 also functions within a heterodimer together with the recently identified metazoan Spo7 orthologous Nuclear Envelope Phosphatase 1-Regulatory Subunit 1 (NEP1-R1; aka TMEM188) to activate lipin-1 [52]. It will therefore be interesting to evaluate whether the CTDNEP1-NEP1-R1 heterodimer may also control lipin-1 function in response to TORC1 in higher eukaryotes. Such insight may prove valuable for our understanding of diseases that are associated with deregulated lipin-1 function such as lipodystrophy, peripheral neuropathy, and insulin resistance in mice, or recurrent osteomyelitis and rhabdomyolysis in humans [8], [53].
